# Electrolytes assessed by point-of-care testing – Are the values comparable with results obtained from the central laboratory?

**DOI:** 10.4103/0972-5229.78219

**Published:** 2011

**Authors:** Binila Chacko, John V Peter, Shalom Patole, Jude J Fleming, Ratnasamy Selvakumar

**Affiliations:** **From:** Medical ICU, Christian Medical College & Hospital, Vellore, India; 1 Department of Biochemistry, Christian Medical College & Hospital, Vellore, India; 2 Department of Clinical Biochemistry, Christian Medical College & Hospital, Vellore, India

**Keywords:** Agreement, bland and altman, concordance, electrolytes, point-of-care testing

## Abstract

**Background and Aims:**

When dealing with very sick patients, the speed and accuracy of tests to detect metabolic derangements is very important. We evaluated if there was agreement between whole blood electrolytes measured by a point-of-care device and serum electrolytes measured using indirect ion-selective electrodes.

**Materials and Methods:**

In this prospective study, electrolytes were analyzed in 44 paired samples drawn from critically ill patients. Whole blood electrolytes were analyzed using a point-of-care blood gas analyzer and serum electrolytes were analyzed in the central laboratory on samples transported through a rapid transit pneumatic system. Agreement was summarized by the mean difference with 95% limits of agreement (LOA) and Lin’s concordance correlation (*p* _c_).

**Results:**

There was a significant difference in the mean (±standard deviation) sodium value between whole blood and serum samples (135.8 ± 5.7 mmol/L vs. 139.9 ± 5.4 mmol/L, *P* < 0.001), with the agreement being modest (*p*_c_ = 0.71; mean difference −4.0; 95% LOA −8.78 to 0.65). Although the agreement between whole blood and serum potassium was good (*p*_c_ = 0.96), and the average difference small (−0.3; 95% LOA −0.72 to 0.13), individual differences were clinically significant, particularly at lower potassium values. For potassium values <3.0 mmol/L, the concordance was low (*p*_c_ = 0.53) and the LOA was wide (1.0 to −0.13). The concordance for potassium was good (*p*_c_ = 0.96) for values ≥3.0 (mean difference −0.2; 95% LOA −0.48 to 0.06).

**Conclusions:**

Clinicians should be aware of the difference between whole blood and serum electrolytes, particularly when urgent samples are tested at point of care and routine follow-up electrolytes are sent to the central laboratory. A correction factor needs to be determined at each center.

## Introduction

Electrolyte abnormalities can precipitate life-threatening events. In such situations, rapid and accurate assessment of electrolyte abnormalities may enable the institution of focused therapies. The rapidity of such assessment, particularly in developing countries, is often limited by the delay in transporting samples to the central laboratory, either due to lack of sufficient numbers of human couriers or the absence of rapid transit systems (RTS). This often results in a long turnaround time (usually over 15 minutes)[1] for the measurement of electrolytes in central laboratories. These delays could unfavorably impact outcomes. Point-of-care testing (POCT) has thus been increasingly used in the emergency department (ED) and the intensive care unit (ICU) to enable the rapid assessment of electrolyte and arterial blood gas (ABG) abnormalities.

Despite the advantage of a rapid turnaround time with POCT, that may translate to prompt decision making, concerns have been raised regarding the accuracy and reliability of POCT devices. Conflicting results from various studies, probably due to the use of different devices, add to these concerns. Whilst some studies concluded that results differed significantly for plasma sodium and chloride concentrations, others also found significant differences in potassium values.[2,3] Thus, it is not uncommon to find clinicians using the POCT results to act in an emergent situation (particularly where extremes of electrolyte values are obtained) whilst sending an additional sample to the central laboratory to “confirm” the POCT values. The differences in the values obtained have been attributed to the use of different devices, the effect of transport of samples through a pneumatic system as well as the type of sample used.

This prospective study was thus undertaken to assess the correlation between whole blood electrolytes measured by a point-of-care device and serum electrolytes measured at a central laboratory, of patients in the ICU. Whole blood electrolyte estimation at the point of care and serum electrolyte estimation at the central laboratory were chosen as the comparators since these reflect practice in most hospitals (whole blood is used at POCT and serum sample at the central laboratory). We also quantified the magnitude of difference between these two estimations, since such an estimate would provide the clinician with a “correction factor” that could be applied to point-of-care values. Although *pre hoc* we did not intend to explore the reasons for discrepancy between the values obtained from the two measurements, we attempted to describe possible factors that contributed to the discrepancy in results following the conclusion of the study.

## Materials and Methods

The study was performed from the medical ICU of a 2200-bed tertiary care university affiliated hospital in India. This ICU is a 12-bedded Level 3 critical care unit manned by four full-time intensivists, residents rostered on-site around the clock in addition to nursing, paramedical staff and respiratory technicians. The study cohort consisted of a convenience sample of 44 samples drawn from patients who required ABG estimation. From each patient, two samples of arterial blood were collected at the same time. The first sample of 1.6 mL was collected from the arterial line in commercially available plastic ABG syringes (DRIHEP A-LINE arterial blood gas collection syringe, 3.0 mL volume, 1.6 mL recommended draw Becton Dickinson Diagnostics^®^, Plymouth, UK) coated with lithium-heparin. The second sample was collected in a BD vacutainer for serum (4.0 mL or 6.0 mL) in a non-additive silicone coated tube, and sent to the central laboratory through the pneumatic system for serum electrolytes’ estimation.

The ABG (whole blood) electrolytes were estimated on-site immediately after collection, using a GEM 3000^®^ ABG analyzer that has direct ion-selective electrodes. Day-to-day precision of the GEM Premier 3000 for aqueous quality control materials [% coefficient of variation (%CV)] was sodium: 0.4–1.2% and potassium: 0–1.3%.[4] Serum electrolytes were analyzed in the central laboratory using an Olympus AU2700 discrete chemistry analyzer (Olympus Optical Company, Ltd., Japan). This chemistry analyzer uses indirect ion-selective electrodes and pre-dilutes the specimen before analysis. Using daily Quality Control (QC) values (BioRad, Lyphochek Assayed Chemistry Control level and Roche Precinorm U), for a mean serum sodium value of 121 mmol/L, the between day coefficient of variation is 1.4%, whilst for a mean sodium of 125 mmol/L, it is 1.6%. For potassium mean values of 3.5 mmol/L, the between day co-efficient of variation is 3.3%, and for a mean potassium of 5.9 mmol/L, it is 2.6%. The study was funded by the Clinical Biochemistry Department of the hospital.

### 

#### Statistical aspects

Agreement was summarized by the mean difference with Bland and Altman’s 95% limits of agreement (LOA).[5] Lin’s concordance correlation (*p*_c_) which describes the relationship between paired measurements was also used.[6] As opposed to Pearson correlation, which measures the strength of a linear relationship but may not pass through the origin and have slope not equal to unity, *p*_c_ compares agreement (between two sets of measurement) by assessing the variation from the 45° line through the origin.

## Results

**Figure 1:**
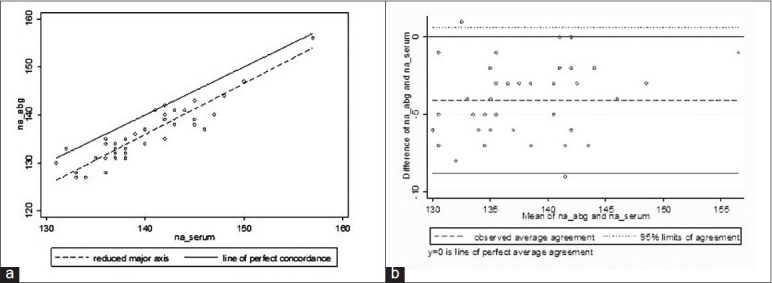
Relationship between serum sodium and whole blood sodium values analyzed for 44 sodium values. (a) Concordance plot showing agreement between serum and whole blood sodium samples (in mmol/L). The solid line indicates the line of perfect concordance and the dotted line indicates the best fitted line for our data. (b) Bland and Altman plot depicting the agreement between serum and whole blood values with 95% LOA (small dotted lines) and the observed average agreement (bold dotted lines). The bold line at y = 0 indicates the line of perfect average agreement

Forty-four paired samples were collected. There was a significant difference in the mean (SD) sodium value between whole blood and serum samples (135.8 ± 5.7 mmol/L vs. 139.9 ± 5.4 mmol/L, *P* < 0.001). The agreement between the two (*p*_c_ = 0.71; mean difference −4.07; 95% LOA −8.8 to 0.7) was modest [Figure 1].

**Figure 2:**
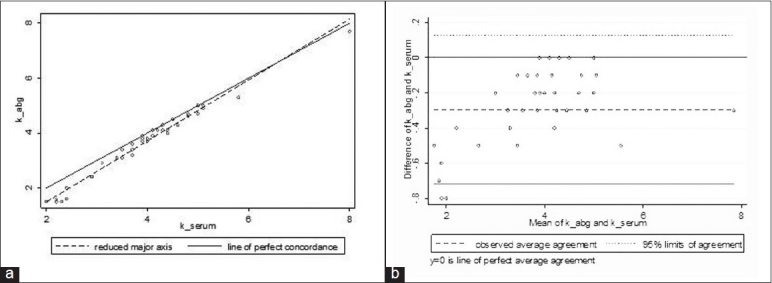
Relationship between serum potassium and whole blood potassium. (a) Concordance plot showing agreement between serum and whole blood potassium samples (in mmol/L). The solid line indicates the line of perfect concordance and the dotted line indicates the best fitted line for our data. (b) Bland and Altman plot depicting the agreement between serum and whole blood values with 95% LOA (small dotted lines) and the observed average agreement (bold dotted lines).The bold line at y = 0 indicates the line of perfect average agreement

**Figure 3:**
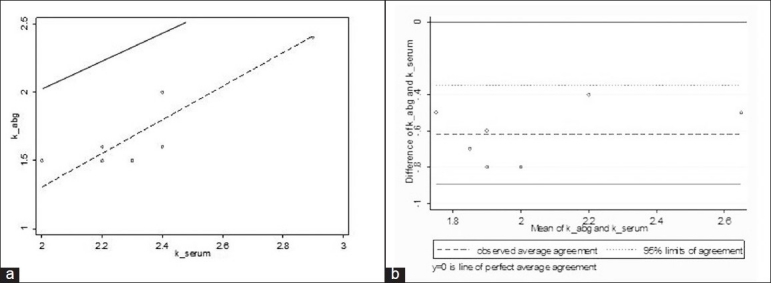
Relationship between serum potassium and whole blood potassium analyzed for 11 potassium values <3 mmol/L. (a) Concordance plot showing agreement between serum and whole blood potassium samples <3mmol/L. The solid line indicates the line of perfect concordance and the dotted line indicates the best fitted line for our data. (b) Bland and Altman plot depicting the agreement between serum and whole blood values with 95% LOA (small dotted lines) and the observed average agreement (bold dotted lines).The bold line at y = 0 indicates the line of perfect average agreement

Analysis of the potassium results obtained by the two methods revealed interesting findings. Although the agreement between whole blood and serum potassium was good (*p*_c_ = 0.96) [Figure 2], and the average difference small (−0.3; 95% LOA −0.72 to 0.13), individual differences were clinically significant, particularly at lower potassium values. For potassium values <3.0 mmol/L, the concordance was low (*p*_c_ = 0.53) and the LOA was wide (−1.0 to −0.13) [Figure 3]. The concordance for potassium for values ≥3.0 mmol/L (mean difference −0.2; 95% LOA −0.48 to 0.06) was good (*p*_c_ = 0.96).

A correction factor for sodium and potassium was estimated. This is shown as the mean difference in the table [Table 1]. For sodium, the correction factor between whole blood and serum was 4 mmol/L. For values of potassium <3 mmol/L, the correction factor was 0.6 mmol/L, and it was 0.2 mmol/L for values above 3 mmol/L.

**Table 1:** Concordance between arterial blood gas and serum electrolyte values

Electrolyte	No. of ABG samples*	ABG value (mmol/L) Mean ± SD	No. of serum samples*	Serum value (mmol/L) Mean ± SD	pc	Mean difference	95% LOA	*P* value
Sodium level	44	135.8 ± 5.7	44	139.9 ± 5.4	0.71	−4.0	−8.78 to 0.65	<0.001
Sodium < 130 mmol/L	4	127.5 ± 0.58	0	—	—	—	—	—
Sodium 130–145 mmol/L	38	135.8 ± 4.0	39	138.6 ± 4.1	0.59	−4.0	−8.6 to 0.6	<0.001
Sodium > 145 mmol/L	2	151.5 ± 6.4	5	149.6 ± 4.4	0.62	−4.8	−11.1 to 1.5	0.007
Potassium level	44	3.6 ± 1.3	44	3.9 ± 1.1	0.96	−0.3	−0.72 to 0.13	<0.001
Potassium < 3 mmol/L	11	1.9 ± 0.6	9	2.3 ± 0.3	0.53	−0.6	−1.00 to −0.13	<0.001
Potassium 3–4.0 mmol/L	15	3.6 ± 0.3	15	3.7 ± 0.3	0.63	−0.2	−0.51 to 0.03	<0.001
Potassium > 4 mmol/L	18	4.7 ± 0.8	20	4.9 ± 0.9	0.96	−0.2	−0.47 to 0.09	<0.001
Potassium > 5 mmol/L	2	6.5 ± 1.7	4	6.0 ± 1.5	0.97	−0.3	−0.60 to 0.60	<0.001

*p*_c_, Lin’s concordance correlation; SD, standard deviation; LOA, limits of agreement,

* The number of samples within a specific range is different for the ABG sample and the serum sample as the paired values may not lie in the same range when assessed by different methods

## Discussion

This study has demonstrated a clinically significant difference between electrolyte values obtained from serum samples estimated at a central laboratory and values obtained from whole blood samples estimated at point of care. Although this difference between whole blood and serum electrolyte estimations is described[7] and known for a long time, there are several aspects to this observation which make this study important in the current day critical care milieu. Our study demonstrated that concordance between serum and whole blood electrolytes may not be uniform across the entire range of values. We observed that whilst concordance was good for potassium values >3 mmol/L, the differences were large for values below 3 mmol/L. The difference for sodium values, on the other hand, appeared uniform across the range [Figure 1].

These results have implications in clinical practice. It is not uncommon to act on ABG electrolyte values and subsequently send a follow-up electrolyte sample to the laboratory to assess the effect of the intervention. This intervention may be in the form of replacement of potassium based on an ABG value or it may be the initiation of measures to reduce potassium levels where a very high value was obtained. Following the initial treatment based on the ABG values, the repeat sample following intervention is often sent to the central laboratory. If the results of this study are applied, then it would appear that the magnitude of correction of potassium would be exaggerated by the fact that whole blood potassium values can be lower by up to 1 mmol/L in ranges below 3.0 mmol/L.

It is likely that the difference of electrolyte values in the paired samples is due to the difference in the type of sample – serum or whole blood. It is well known that potassium is released from the platelets during the clotting process,[8] and thus, it is not surprising that the serum potassium values are higher than whole blood potassium values. However, the magnitude of this difference in our study appeared higher than what is reported in literature (0.1–0.7 mmol/L).[7] Hence, we looked for other factors that might explain the exaggerated difference between the whole blood and serum values. These include transport of samples through a rapid transit system, variations due to dilution of serum samples prior to testing (indirect vs. direct electrodes) as well as variations due to the calibrator used in each instrument. These are discussed below.

Studies that have evaluated the impact of transport of samples through the pneumatic system on electrolytes values have suggested that RTS does not significantly change serum electrolytes values.[9] In a quality assurance project at the time of installation of our pneumatic system, we observed that transport of samples through the RTS did not significantly alter the electrolyte values when the type of sample and the type of analyzer were similar. Thus, the difference between the two samples is unlikely to be due to transport of the sample through the pneumatic system.

The type of electrodes used for analysis could have also influenced the magnitude of difference between the two estimations. The POCT device uses the direct ion-selective electrode, and the Olympus analyzer in the central laboratory for analysis of serum electrolytes uses the indirect ion-selective electrode. Direct ion-selective electrodes measure the activity of ions in the plasma water, which is directly proportional to their concentration, whilst indirect ion-selective electrodes measure the activity of ions in a pre-diluted sample. Indirect ion-sensitive electrodes are affected by dissolved solids, such as proteins, in the sample and this factor could have also influenced the values obtained by the two methods.

Finally, the two instruments used in this study have different calibrators and this could have also contributed to the differences in this as well as in the previous studies on this aspect.

Thus, the observed difference between serum and whole blood electrolytes is due to a combination of factors that include the type of sample (whole blood vs. serum), variations in the calibration of the machine as well as dilution of the serum sample. Variations as a result of the above factors can be compensated using a correction factor. Although a correction factor may result in off-shoot (overestimate or underestimate) of values in some situations, its use is to minimize the differences between the analyzers by making an average compensation.[10] The concept of application of a correction factor is not new. The International Federation of Clinical Chemistry recommends a constant conversion factor (of 1.11) between whole blood and plasma glucose to provide “harmonized results”, facilitating the classification and care of patients and leading to fewer therapeutic misjudgements.[10] However, the correction factor for electrolytes may need to be generated for each center.

Although there are other studies on this topic,[1–4,11] our study is different in the following ways: (a) in contrast to an earlier study[11] in which both samples (the whole blood and the serum) were analyzed in the central laboratory; in our study, the whole blood sample was analyzed at the point of care (which is the standard practice) and the serum sample was analyzed in the central laboratory; (b) we used paired arterial samples drawn at the same time rather than using[11] an arterial sample for the POCT and a venous sample for the laboratory testing; (c) although one study[4] used the same machine as ours for POCT (i.e. GEM 3000^®^ ABG analyzer), other studies have evaluated the differences using other machines.[11] The samples that were analyzed in the central laboratory were transported through a pneumatic system in our study, which is not a feature of other studies. Further, in order to minimize the effect of dilution of the sample by using heparin flushes in the syringes, we used plastic lithium-coated ABG syringes. The statistical tools that were used in our study to compare the two methods were also more appropriate than simple comparisons using the paired *t*-test.

This study, however, needs to be interpreted in the light of the following limitations. The sample size was small. Although data for potassium were available across the range for potassium values, there were very few sodium values in the hypernatremic and hyponatremic ranges. This limits the application of correction factors for sodium, particularly in the abnormal ranges in our patient population where evidence suggests that there could be marked discordance at lower sodium values.[11]

## Conclusions

This study illustrates the importance of determining the concordance between electrolyte values obtained by POCT and those obtained in the central laboratory for each individual hospital. Since all these factors may differ from place to place (e.g. type of instrument, the calibration, etc.), it is important that each center does its own study and derives a correction factor that may need to be applied if different methods of estimation are used. The correction factor in our study was 4 mmol/L for sodium, 0.6 mmol/L for potassium values <3 mmol/L and 0.2 mmol/L for values 3 and above.
